# Human papillomavirus vaccination uptake and its associated factors among adolescent school girls in Ambo town, Oromia region, Ethiopia, 2020

**DOI:** 10.1371/journal.pone.0271237

**Published:** 2022-07-13

**Authors:** Mulugeta W/mariam Beyen, Gizachew Abdissa Bulto, Eshetu Ejeta Chaka, Bikila Tefera Debelo, Ephrem Yohannes Roga, Negash Wakgari, Kababa Temesgen Danusa, Daniel Belema Fekene

**Affiliations:** 1 South West Shewa Zone Health Office, Waliso, Ethiopia; 2 Department of Midwifery, College of Medicine and Health Science, Ambo University, Ambo, Ethiopia; 3 Epidemiology and Biostatistics Unit, Department of Public Health, College of Medicine and Health Science, Ambo University, Ambo, Ethiopia; Baylor College of Medicine, UNITED STATES

## Abstract

**Background:**

The Human Papillomavirus (HPV) vaccine has offered a great promise to reduce the cervical cancer burden; its utilization (uptake) however has been lagging. However, the levels and factors associated with the uptake of the vaccine have not been well investigated, especially in the local context.

**Objective:**

To assess the uptake of human papillomavirus vaccination and its associated factors among adolescent school girls in ambo town, Oromia, Ethiopia, 2020.

**Methods:**

An institution-based cross-sectional quantitative study design supplemented with the qualitative inquiry was employed to assess Human Papillomavirus vaccination uptake and its associated factors among 422 adolescent school girls in Ambo town, central Ethiopia from December 1–30, 2020. The collected data were coded, entered, and cleaned by using Epi info 7.2.3 and exported to SPSS version 25 for analysis. Descriptive statistics were used to compute summary statistics and proportions. Both bivariate and multivariable logistic regression was employed to identify factors associated with HPV vaccine uptake. Adjusted odds ratio and 95% confidence interval were used for the strength and directions of association. A P-value of < 0.05 was used to declare statistical significance. Qualitative findings have been analyzed with manual thematic analysis.

**Result:**

The proportion of HPV vaccination uptake among school girls in this study was 44.4%. Hearing about HPV vaccine [AOR = 2.50, 95%CI: (1.045–5.959)], availability of awareness creation [AOR = 2.53, 95%CI: (1.507–4.258)], and favorable attitude [AOR = 2.049, 95%CI: (1.153–3.64)] were the key identified factors associated with vaccination uptake. In addition, poor perception, fear of side effects, and misunderstanding were among the major factors identified by qualitative findings.

**Conclusion:**

There was low uptake of HPV vaccination among the school Adolescents in the study area. Availability of awareness creation programs, favorable attitude towards HPV vaccine, and hearing about HPV vaccine was significantly associated with the uptake of the HPV vaccination. Therefore, awareness creation and behavior change education are mandatory to scale up the vaccination.

## Introduction

Cervical cancer is the fourth most common cancer affecting women and accounts for about 7.5% of cancer-related deaths in women globally [1]. More than 99% of cervical cancer is caused by persistent infection with the oncogenic or high-risk sexually transmitted types of Human Papilloma Virus (HPV) [2]. One woman dies of cervical cancer every two minutes, affecting about 570,000 women with an estimated 311,000 deaths across the globe, greater than 85% of the deaths occurring in less developed countries [3, 4]. Many of the victims of the disease are young, undereducated women living in the world’s poorest countries where access to pre-screening and treatment is limited, patients seek medical care after a complication occurred at an advanced stage and a majority of them do not have access to prevention programs/services [3, 5].

However, it’s a preventable form of cancer through the prevention of human papillomavirus (HPV) infection [6]. This can be achieved by the highly effective and cost-effective prevention strategy using the HPV vaccine which prevents the commonest strains of high-risk and low-risk human papillomavirus infection [7]. As of 2020, more than half of the WHO member countries have introduced HPV vaccination programs to meet the 2030 Sustainable Development Goal (SDG) elimination target of 90% [8]. Studies reported as high as 83% reduction of HPV 16 and 18 infections among girls aged 13–19 years after up to eight years of follow-up post-vaccination [9]. A single dose of HPV vaccine offers similar protection against high-risk strains of HPV (types 16, 18) as two or three doses of HPV vaccination [10]. It has the potential to avoid 70% of cervical cancers and will be an important element of a cervical cancer control strategy [5, 6]. Ensuring universal access to HPV vaccination will be key to reducing the burden of cervical cancer worldwide [11].

Since its introduction, many countries have tried to implement school-based immunization programs which were remarkably successful in increasing the adolescents’ vaccination rate. Studies showed that HPV-vaccine uptake levels (at least one dose) varied significantly among countries as low as 1.1% to 94.4%. The review study shows that high-income countries such as Scotland and Taiwan that have successful programs have reached >80% [12]. However, many sub-Saharan African countries that had been delayed in HPV-Vaccine introduction were still having low coverage [13], in Uganda for instance, HPV vaccine uptake was 17.61% whereas, in contrast, the prevalence of cervical cancer was high [14]. In Ethiopia, about 7,445 new cervical cancer cases are diagnosed and about 5,338 women die of it annually [15]. In Ethiopia since its introduction in 2018, the vaccination program is restricted only to a single cohort of 14 years (only) and lacks any (published) standard report that indicates the status of coverage level [12, 16].

However, its acceptance by the public has been lagging for several reasons [7]. To explore these reasons, studies have looked at hindering factors at the community level (social group values and norms, media coverage around the HPV vaccine), at the organizational level (allocated resources, information provision, consent process, immunization setting, and environment) and the policy level (HPV vaccine program) [3, 4, 17–20]. Additionally, poor knowledge about cervical cancer, HPV infection, HPV vaccine, negative attitude towards the HPV vaccine, poorly perceived susceptibility and severity of cervical cancer age, residence, level of education, parental educational status, and low socioeconomic status, significantly affects the vaccine uptake [3, 4, 11, 13, 17–32].

WHO set strategy design and coverage improvement plans to reduce the burden of the disease and to reach the 90% goal of the global cervical cancer elimination program. To address such a strategy, different studies need to be conducted pre-and-post the deliverance of the vaccine expecting a high contribution to the success of the program. Therefore, an evidence-based study on the HPV vaccine and associated factors is a cornerstone for tracking the progress of the program to eliminate diseases caused by HPV and achieve the stated goal. However, in Ethiopia, the vaccine is newly introduced and studies conducted to analyze the vaccine uptake level and its associated factors are very limited. Therefore, this study might be used to identify barriers and factors affecting the utilization of the HPV vaccine which might be used as basic information for further interventions. Additionally, this study aimed at providing important information for policymakers and program implementers at national as well as regional levels, and the information derived from the study considered it would benefit the adolescent by providing directions on service provision. Therefore, this study assessed the uptake of HPV vaccination and its associated factors among adolescent school girls in Ambo town, Oromia, Ethiopia.

## Methods

### Study setting, design, and population

An institution-based cross-sectional quantitative study design supplemented with the qualitative inquiry was employed to assess HPV vaccination uptake and its associated factors among Adolescent school girls in Ambo town, central Ethiopia from December 1–30, 2020. The qualitative inquiry was included to describe various aspects of behaviors, feelings, perceptions, and experiences related to the HPV vaccination program. Ambo town has sixteen primary schools of which nine are governmental and seven private schools and a total of five secondary schools of which four are governmental and one private school. The school-based registered number of adolescent girls aged 10–19 years among all government and private schools was 10,091. Based on the Ambo town health office report, the first cycle of HPV vaccination has been initiated with a campaign in all primary schools for only 14-year-old girls since 2018.

The study was complemented by qualitative inquiry to explore in detail cultural, behavioral, and social factors that might affect HPV vaccine uptake. Accordingly, health professionals working on the HPV vaccination program at Ambo town health offices and health facilities, school teachers who were involved in HPV vaccination program in a town, and adolescent girls aged 14–18 years in schools who have participated in different health clubs (like Reproductive Health, HIV-Club, Red Cross association members, environmental sanitation) and purposively selected community leaders were interviewed during the qualitative inquiry.

### Sample size determination and sampling procedure

The sample size for the quantitative study was determined by using single population proportion formula with a prevalence (p) estimate of HPV-vaccine 50%, 5% of marginal error (d), and 95% confidence level. Finally, by considering 10% as a non-response rate the calculated final sample size was 422.

**Fig 1:**
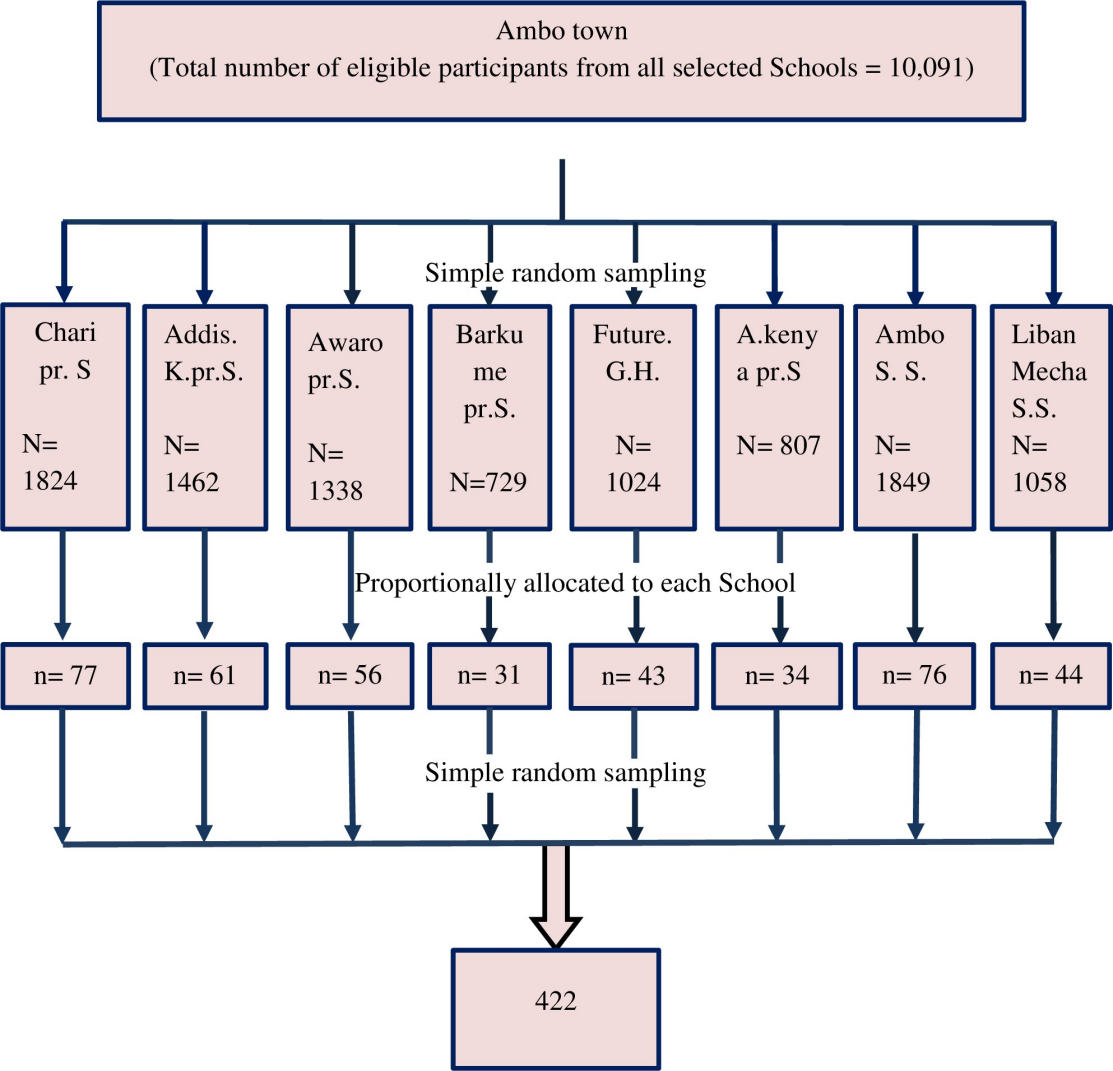
Schematic presentation of the sampling procedure on uptake of human papillomavirus vaccination and its associated factors among adolescent school girls in Ambo town, central Ethiopia, 2020. Gr-Grade, Pr- primary school, S.S–Secondary School.

To select the study participants in the quantitative approach, six out of 16 primary schools and two out of five secondary schools in Ambo town were selected by simple random sampling using a lottery method. For each selected school sample size was determined proportionally according to the number of adolescent girls within the school. The study participant was selected from each school using simple random sampling by lottery method **(Fig 1)**. For the qualitative data, purposively selected key informants consisting of health professionals, community leaders, community volunteer workers, and teachers were organized where the study subjects gave their views on the questions in the key informant interview guide. In addition, some adolescent girls were selected for an in-depth interview.

### Data collection tools and techniques

Structured questionnaires were prepared in English, translated into Afan Oromo, and then translated back into English to check for consistency. The questionnaires were adapted through reviewing different literature and previous similar studies.

The questionnaire tool has eight sections that were developed to assess socio-demographic characteristics, knowledge about cervical cancer, HPV infection and vaccination, perceptions, attitudes towards HPV vaccination, presence of promotion, and sources of information about HPV vaccination and HPV vaccine uptake. Data was collected using self -administered Afan Oromo version questionnaires. To administer the structured questionnaire eight teachers as data facilitators- one for each selected School (experienced data collector) and three Nurses as supervisors were recruited. The training was given for one day on the objective, relevance of the study, confidentiality of information, respondent rights, informed consent, and techniques of interview.

For the qualitative data, open-ended questions were prepared by focusing on the types of information required from the participant. Data were collected through an in-depth interview. One interviewer for each in-depth interview was recruited to conduct the interview. An in-depth interview was conducted for each category of adolescents who had not received, received one, and received two HPV vaccinations respectively, and a key informant interview (KII) was conducted on those who know the issue of the concern, an expert opinion, and can give a variety of perspectives about HPV vaccination. The interview had been recorded in tapes which were later transcribed.

### Data quality control

For the quantitative approach, to maintain the quality of the data, data collectors and supervisors were trained in data collection procedures. Before actual data collection time, the questionnaire (tool) was pretested for validity and reliability on 5% of the sample in schools that were not selected for the actual data collection to assess instrument simplicity, flow, and consistency; thereby possible adjustment or modification was considered/ on a time of deliverance and little correction on some questions had been incorporated. The principal investigator and supervisors were made spot-checking and reviewing all the completed questionnaires to ensure completeness and consistency of the information collected. The data collectors were supervised by three supervisors. Data entry has been done by the principal investigator to keep the accuracy of the data.

For the qualitative approach, data quality was ensured by enhancing the trustworthiness and rigor of the study findings. The in-depth interview record and note-taking were transcribed immediately after the interview was conducted. The recorded tapes were listened and transcriptions were read and re-read several times carefully. From the potentially important and provisional notes, the researcher noted the interrelationships, connections, and patterns that emerged in the data. These were developed into themes.

### Data processing and analysis

The quantitative data were checked for completeness, and consistency of the information obtained from the respondent and entered into Epi info version 7.2.2.6. Then data were exported to SPSS version 25 for cleaning, editing, and analysis. The data were checked for missed values and outliers. Descriptive analysis (like frequencies, tables, percentages, means, and standard deviation) was done to describe variables. Both bivariate and multivariable logistic regressions were performed to identify significant factors associated with HPV vaccine uptake. Those variables with p<0.25 in bivariate analysis were considered for multivariable logistic regression analysis. Multivariable logistic regression at the 95% confidence level was used to identify the predictors. A significance level of 0.05 was taken as a cutoff value for all statistical significance tests. Multi-collinearity was detected between predictors when checked using the variance inflation factor for each variable. Accordingly, two variables that yield 9.95 and 11.5 were removed on Multivariable logistic analyses (VIF = 9.95 & 11.5).

Data from an in-depth interview were audiotaped and recorded then transcribed verbatim, then coded, arranged by themes, and analyzed thematically. Tape-recorded and note obtained from the in-depth interview were transcribed carefully by investigators into Afan Oromo language word by word and arranged with the written notes taken at the time of the interview. The information was translated into English. Thematic content analysis was employed to describe the ideas obtained from the in-depth interview. Through this process, a verbatim quotation was used to illustrate responses on relevant issues and themes. Finally, it was incorporated with the quantitative findings to provide comprehensive and complete information.

### Measurements

In this study, uptake of the HPV vaccine was defined as the proportion of those Adolescents girls who had received at least one dose of the HPV vaccination [12]. A good overall Knowledge of Cervical cancer, HPV infection, and HPV vaccine was measured by asking 16 knowledge-related questions and a score greater than the mean was classified as good knowledge [14]. Additionally, a positive attitude towards HPV vaccination was determined by using seven attitude-related questions with a five-point Likert scale and participants with scores above the mean were classified as having a ‘positive attitude’ towards HPV vaccination [33]. Awareness creation in the present study is the presence of a coordinated information campaign on cervical cancer and HPV vaccination in school by expert professionals or expert trained individuals.

### Ethics statement

Ethical clearance was obtained from Ambo University, College of Medicine and Health Science Research review and ethics committee with the reference number AU/PGC/29/2020. Informed oral consent was obtained from each respondent after explaining the purpose and procedure of the study. Written informed consent was obtained separately from parents to ensure their legal preparation on behalf of their children for adolescents aged 18 and below. To keep the confidentiality of any information provided by study subjects, the data collection procedure was anonymous. Participation was voluntary and withdrawal from the study at any time of data collection was considered. No name or other identifying information was included in the questionnaire.

## Results

### Socio-demographic characteristics of respondents

A total of 414 respondents participated in the study with a response rate of 98.1%. The age of respondents ranged from 14 to 18 years with a mean age of 15.69 and standard deviations of 1.34 years. Regarding parental educational status, more than a third 155 (37.5%) and 183 (44.1%) of the respondent’s fathers and mothers had no formal education respectively **(Table 1)**.

**Table 1:** Socio-demographic characteristics, of adolescent school girls in Ambo town, Ethiopia, 2020 (n = 414).

Socio-demographic characteristics		Frequency	Percent (%)
**Age**	14–15 years	199	48.1
	16–18 years	215	51.9
**Religion**	Orthodox	157	37.9
	Protestant	210	50.7
	Others*	47	11.4
**Fathers’ level of education**	No formal education	155	37.4
	Primary education	107	25.9
	Secondary education	62	15.0
	College Diploma and above	90	21.7
**Mothers’ level of education**	No formal education	183	44.1
	Primary education	112	27.1
	Secondary education	76	18.4
	College diploma and above	43	10.4
**Fathers’ Occupation**	Farmer	198	47.8
	Government employee	118	28.6
	Merchant	42	10.1
	Other**	56	13.5
**Mothers’ occupation**	Farmer	150	36.2
	Housewife	102	24.6
	Government employee	58	14.0
	Merchant	58	14.0
	Other**	46	11.1

*Muslim, Waqeffata, Catholic,

**Prophesier, private employee

### Uptake of the HPV vaccine

**Fig 2:**
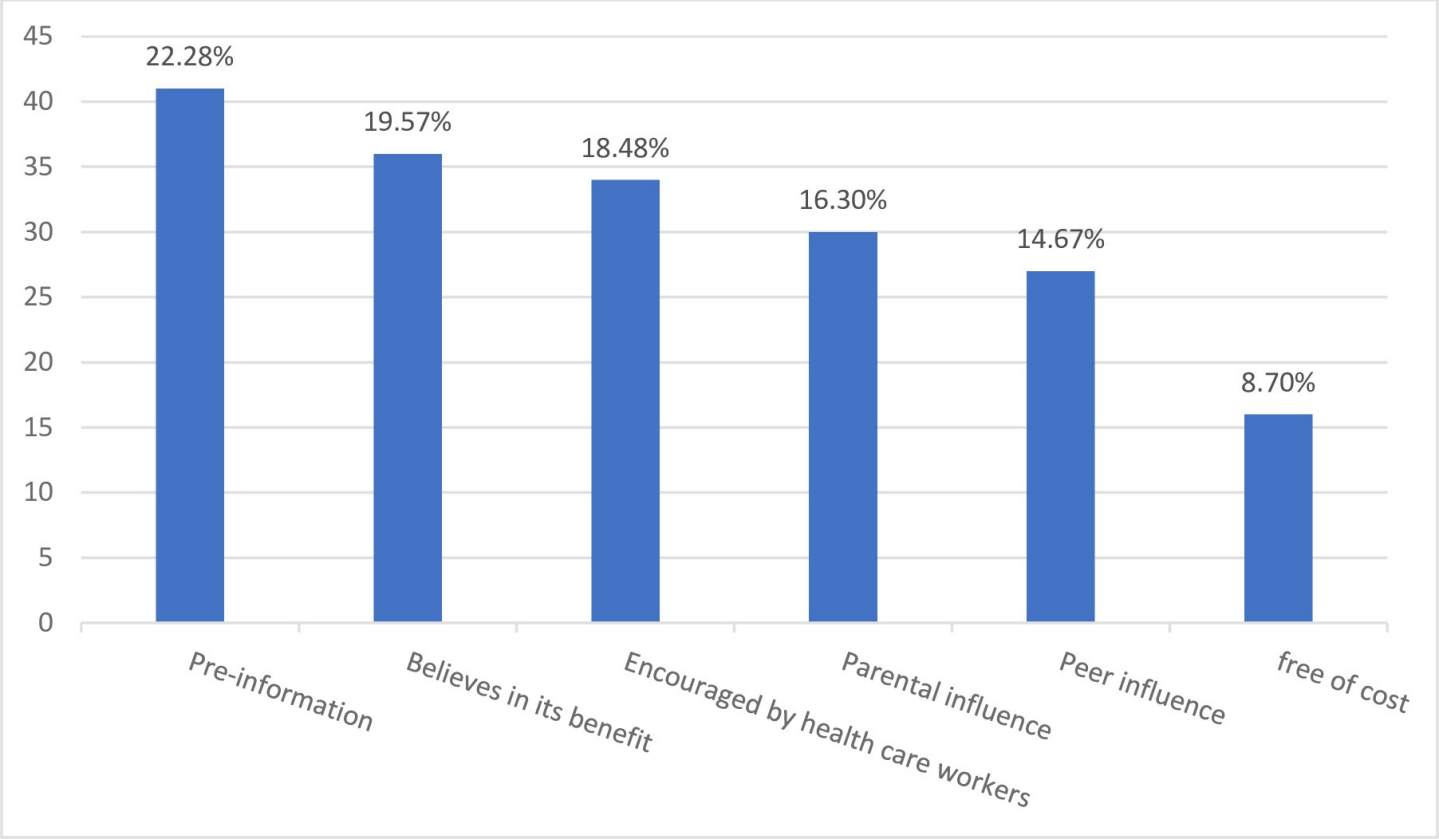
Reasons for considering to get vaccinated among adolescent school girls in Ambo town, central Ethiopia, 2020 (n = 184).

**Fig 3:**
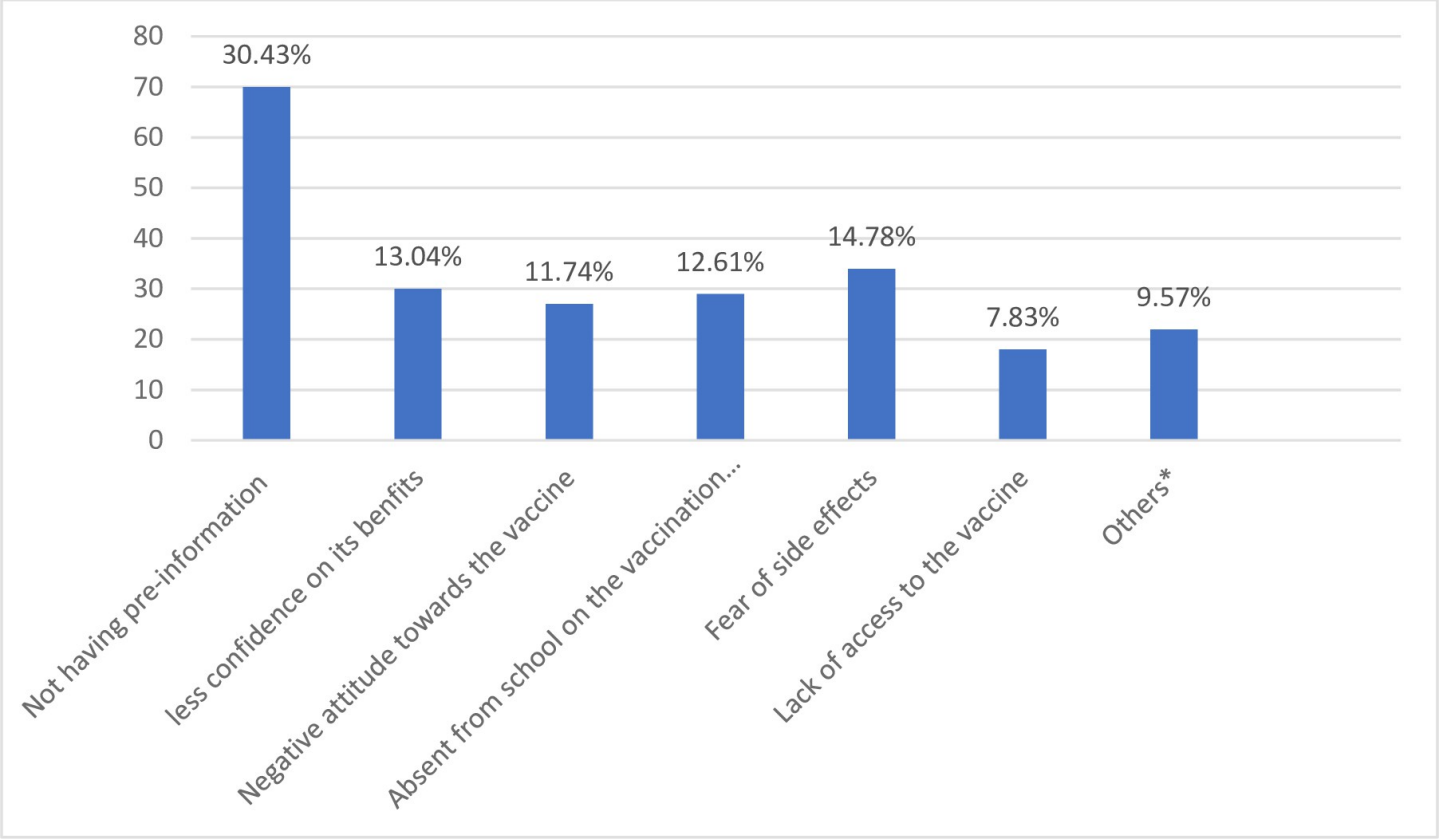
Reasons for not considering to get vaccinated among adolescent school girls in Ambo town, central Ethiopia, 2020 (n = 230). *Parental Influence, peer influence, social pressure.

More than half 230 (55.6%) had not received any dose of the HPV vaccine, 97 (23.4%) had received one dose and 87 (21%) had received two doses. The proportion of the HPV vaccine uptake in the study area was 44.4%. The main reasons mentioned by the respondents for being vaccinated were having pre-information 41(22.28%), believing that it has benefits 36(19.57%), and encouragement from health workers 34(18.48%) **(Fig 2)**. On the other hand, unavailability of pre-information 70(30.4%) fear of side effects 34(14.78%), and being absent during vaccination day 29(12.61%) were some of the reasons for hesitating to get vaccinated among unvaccinated school girls **(Fig 3)**.

However, the interview identified some thought-provoking factors related to HPV vaccination that were left unidentified by the quantitative data. There was weak integration while the HPV vaccination program/campaign/ and the absence of a standard program was the main challenging factor for its implementation. There was no regular, coherent, and stable program/schedule. This leads uncomfortably among stakeholders which even affects their attitude. This was assured by some of the health extension workers:

“*Some stakeholders’ unfavorable attitude was another challenge that affects the vaccination program*. *They considered the duty as only given for health care workers*, *valuing (considering) the benefit*, *inability to cooperate*.”

### Barriers and perceptions toward HPV vaccine uptake

Most of the adolescent school girls, 367 (88.6%) had good overall knowledge about Cervical cancer, HPV infection, and the HPV vaccine knowledge. More than half of the adolescent school girls 230 (55.6%) had a favorable attitude toward the HPV vaccine. The qualitative inquiry investigated high perceived severity among adolescents. Health workers explained that illness and deaths associated with cervical cancer witnessed in the communities were an eye-opener to parents that cervical cancer is fatal and its prevention is critical. Having high perceived severity of the disease and any prior experience related to its consequence could increase fear of the disease which had a determinant effect on views of the vaccine. Such exposures ultimately encourage communities to vaccinate their daughters.

On the other hand, the most common individual barriers to vaccination were concerns about side effects. In addition to this, pain associated with vaccination was another concern. Participants with a higher awareness of their low tolerance for pain felt greater anxiety about the intramuscular injection. Moreover, the vaccine was highly misunderstood as some believed that the vaccine was a hoax by the government, meant to reduce population growth. This was supported by the word of a participant who hasn’t been vaccinated:

*“If it is not aimed to reduce the population growth why it has been given for females only*?*” asks the participant of the interview*. *Many of them associate it with reproductive issues considering rumors circulated in society*. *In some places*, *peer influences were highly seen*. *Especially*, *from boys who repetitively influence girls by seeing the drug description/leaflet and pressure the girls not to take the vaccine*. Even some adolescents had a concern as much as: *“I do not trust it since it has been widely spoken that it [HPV vaccine] is to reduce the number of communities*, *especially the number of Oromos and hence I couldn’t be sure on it”*.

### Source of information, the existence of promotion and pre-information on HPV vaccination

**Fig 4:**
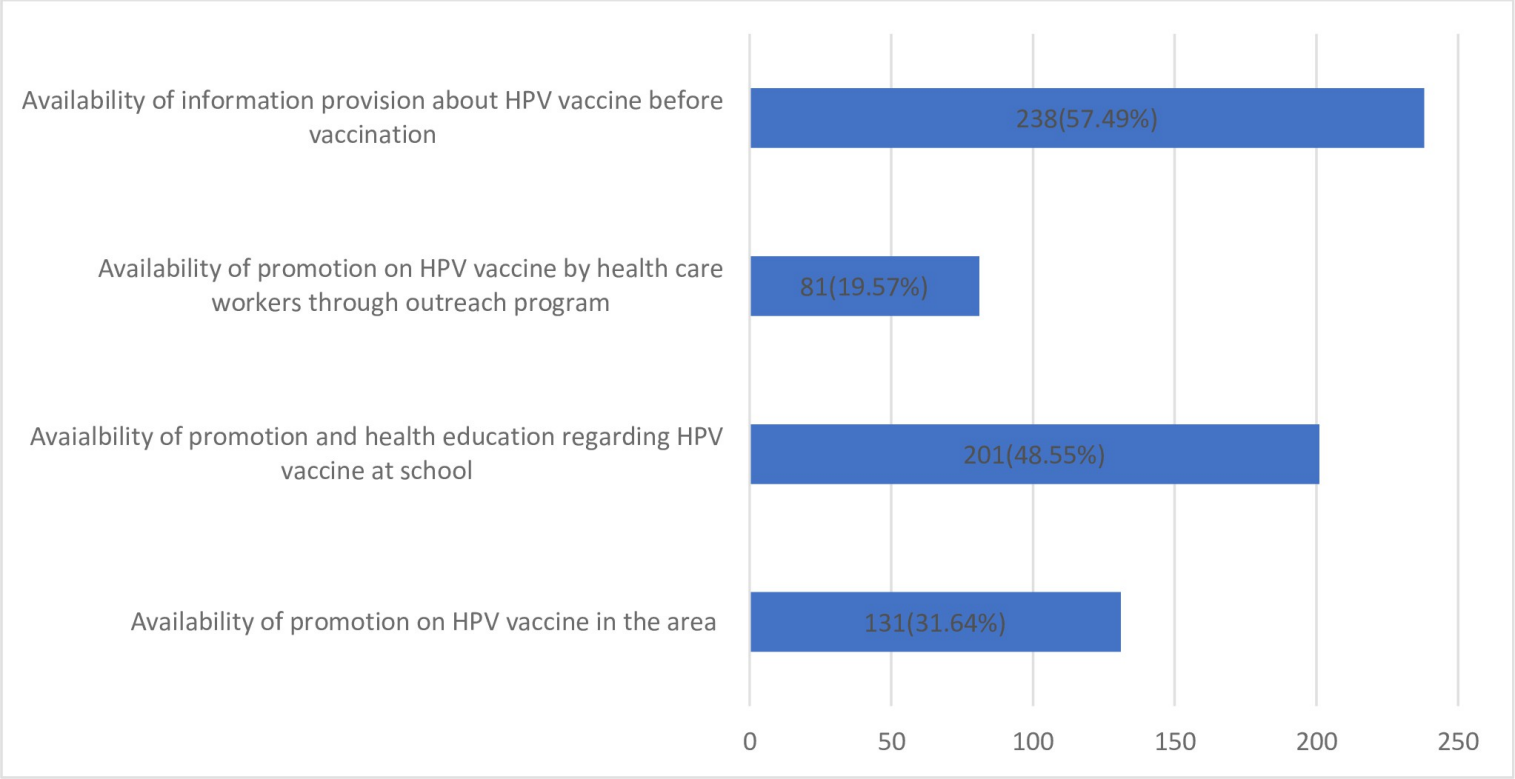
Existence of promotion and pre-information towards the HPV vaccine among adolescent school girls in Ambo town, Ethiopia, 2020 (n = 414).

**Fig 5:**
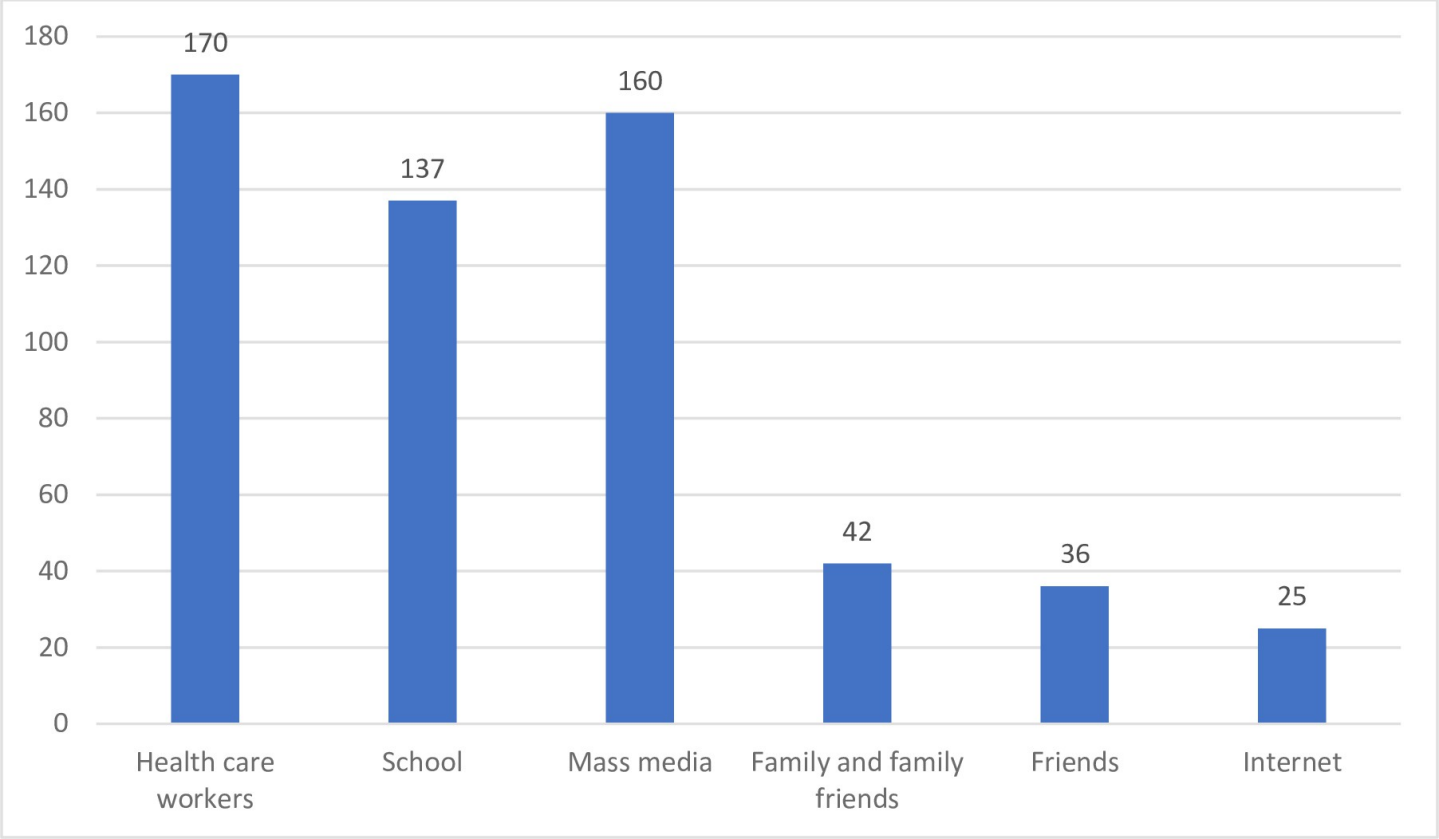
Source of information on HPV vaccination of adolescent school girls in ambo town, central Ethiopia, 2020. Since multiple responses are possible, the percentage could be greater than 100%.

More than half 213 (51.4%) of the adolescent school girls reported the unavailability of promotion and health education regarding the HPV vaccine at school and 333 (80%) of the girls responded that promotion regarding HPV vaccine by health workers through community outreach is lacking. However, 238 (57.5%) of the respondents retorted to the availability of providing full information about HPV vaccines before vaccination **(Fig 4)**. Regarding the potential source of information about the HPV vaccine, health workers 170 (41.06%)and mass media 160 (38.64%)were the main sources of information (**Fig 5**).

In both in-depth interviews and KII, there was a strong expression of social connectedness as a community-level facilitator to promote vaccination. In addition to this, adolescent participants described school-based promotion and vaccination as very important, however, there would less emphasis on promotion and health education. “*We don’t know when we get the vaccine because they gave us leaflet only and told us as we were recommended to be vaccinated without full information*” (*in-depth interview of schoolgirls*).

### Factors associated with uptake of human papillomavirus vaccination

The study also additionally identified the association between uptake of HPV vaccination and some factors or characteristics of the respondent. On bivariate logistic analysis fathers’ level of education, mothers’ occupation, hearing about the vaccine, attitude towards HPV vaccine, availability of promotion in the area, availability of awareness creation about cervical cancer or HPV vaccine at school, and provision full information about HPV vaccine before vaccination were found to be associated with the HPV vaccine uptake.

Multivariable logistic regression analysis revealed that fathers’ level of education, mothers’ occupation, hearing about the vaccine, attitude towards HPV vaccine, and availability of awareness creation about cervical cancer or HPV vaccine at school were significantly associated with uptake of HPV vaccination.

Accordingly, adolescents who have heard about the HPV vaccine before vaccination had the odds of receiving the vaccine 2.5 times higher than those who haven’t heard about it [AOR = 2.50, 95%CI: (1.045–5.959)]. The odds of HPV vaccine uptake were two times higher among adolescents who had a positive attitude toward the vaccine than those who have a negative attitude towards the vaccine [AOR = 2.049, 95%CI: (1.153–3.640)]. Also, the odds of HPV vaccine uptake were 2.5 times higher among adolescent school girls who reported the presence of awareness creation about cervical cancer or HPV vaccine is available at school [AOR = 2.533, 95%CI: (1.507–4.258)] **(Table 2)**.

**Table 2:** Bivariate and multivariable logistic analysis to identify factors associated with uptake of HPV vaccination among adolescent school girls in Ambo town, Ethiopia, 2020 (n = 414).

Variables	Uptake			COR (CI 95%)	AOR (95% CI)	P-value	
	Received	Not received	P-value				
Overall knowledge of Cervical cancer, HPV** infection, and HPV vaccine						
**Good knowledge**	95(23)	73(17.6)	.000	2.296(1.538, 3.428)	.732(.324, 1.654)	.454
**Poor knowledge**	89(21.5)	157(37.9)		1.0	1.0	
**Have you ever heard HPV vaccine**						
**Yes**	171(41.3)	155(37.4)	.000	6.365(3.398, 11.923)	2.495(1.045,5.959)*	.040
**No**	13(3.1)	75(18.2)		1.0	1.0	
**Attitude towards HPV vaccine**						
**Positive**	135(32.6)	104(25.2)	.000	3.338(2.199, 5.067)	2.049(1.153, 3.640)*	. 014
**Negative**	49(11.8)	126(30.4)		1.0	1.0	
**Existence of promotion and pre-information**						
**Is there any available promotion in your area**						
**Yes**	75(18.1)	56(13.5)	.000	2.138(1.403, 3.257)	1.145(.668,1.963)	.623
**No**	109(26.3)	174(42.1)		1.0	1.0	
**Availability of awareness creation about cervical cancer or HPV vaccine at school**						
**Yes**	125(30.2)	76(18.4)	.000	4.293(2.838, 6.494)	2.533(1.507, 4.258)*	< .001
**No**	59(14.1)	154(37.3)		1.0	1.0	
**Do providers provide full information about the HPV vaccine before vaccination**						
**Yes**	119(28.8)	119(28.7)	. 008	1.708(1.147, 2.542)	.892(.536, 1.483)	.660
**No**	65(15.7)	111(26.8)		1.0	1.0	

*statistically significant at p< 0.05 with COR: crude odds ratio AOR, adjusted odds ratio

**HPV- human papillomavirus

## Discussion

Striving for the program coverage and access to the HPV vaccine is very important to decrease the burden of cervical cancer. In this study, the uptake level among the study group was 44.4%. The finding was consistent with a finding from a similar study in the Minakulu sub-county Oyam district, Uganda, in which the uptake was 42.4% [34]. However, this was extensively higher than the study conducted among female Adolescents in selected secondary schools in Ibadan, Nigeria (4.1%) and much lower than the finding of a study done in a developed country, in which the uptake level was much higher in Taiwan (91%) and in Scotland (94.4%) [12, 30]. The difference between the finding and the previous study might be due to the vaccine accessibility; as this country are have better access coverage. They had also implemented routine HPV vaccination for all eligible Girls.

Additionally, the study also tried to investigate factors associated with HPV vaccine uptake. Accordingly, hearing about the vaccine/having pre-information about the vaccine before vaccination was significantly associated with vaccine uptake. The result was in line with the qualitative result from a focused group discussion of a study from the Ibanda District of Uganda in which being exposed to information was associated with HPV vaccination [27]. This might be due to information that helps to cultivate the vaccine benefit. In addition to this, information helps to create a positive attitude toward the vaccine.

On the other hand, the finding of the study indicated that having a positive attitude is associated with higher odds of HPV vaccine uptake. This is supported by the finding in another study conducted in the Liara district of Uganda; in which the uptake of HPV vaccine among adolescents with a positive attitude is three times higher than those with a negative attitude [14]. As well it also realized by a qualitative study conducted in other parts of Uganda (Ibanda district) in which all the focus group discussions depicted almost all vaccinated girls’ had positive attitudes about HPV vaccination [27]. This may be explained by the fact that most motivating/initiative factors of adolescents’ practice are generated from their positive attitude/which can be concluded as the impact of attitude on behavior.

Moreover, the availability of awareness creation about cervical cancer or HPV vaccine specifically at school was associated with the vaccine uptake. This was also supported by qualitative findings; were the respondents described as availability of awareness creation in their school (even if for an irregular/short period) help them to gain more information about the benefits of the vaccine which in turn influenced them to take the vaccine. The result of this study was also consistent with findings from Uganda where they revealed the prevalence of HPV vaccine uptake was higher among adolescents who had been encouraged by a health worker or a village health team member to go for HPV vaccination [14]. This may be because in school availing the information for all can be easily possible and all adolescents have an equal chance to gain important knowledge which possibly increased the rate of vaccination uptake. Another study from Kenya also reported that adequate promotions at the school level were significantly related to uptake level [28].

### Strength and limitation of the study

The study employed a mixed study design to gain detailed data from the research participants. Therefore, the problem has been addressed by both quantitative and qualitative research approaches. On the other hand, the study didn’t address all components of perception which might well be addressed by the health belief model. Also, the study was limited to school facilities, it was difficult to generalize to all adolescents of similar age groups living in the same town.

## Conclusion

In this study finding, having pre-information about the vaccine before vaccination, attitude, and the existence of awareness creation about cervical cancer or HPV vaccine specifically at school were found to be statistically significant with HPV vaccine uptake. The main barriers to HPV vaccination were lack of pre-information, fear of side effects, poor perception, and no regular program or schedule for the immunization. Therefore, regular HPV vaccination schedules and sensitizations should be done to build positive attitudes towards the vaccine which have paramount importance to increasing the uptake. The finding also indicated the need to improve health education programs for adolescent vaccination. Also, to minimize the effect of the limitations mentioned above scholars with similar interests are recommended to conduct a community-based study.

## Supporting information

S1 File Afaan Oromoo version questionnaire for the quantitative approach. (PDF)

S2 File Afaan Oromoo version checklist for the qualitative inquiry. (PDF)

S3 File English version questionnaire for the quantitative approach. (PDF)

S4 File English version checklist for the qualitative inquiry. (PDF)

S5 File Informed parental Ascent form. (PDF)

## List of abbreviations

HPV: Human Papilloma Virus
HIV: Human Immune-deficiency Virus
