# Supplementary material for: Human papillomavirus vaccination uptake and its associated factors among adolescent school girls in Ambo town, Oromia region, Ethiopia, 2020
Source: PLoS One. 2022 Jul 13;17(7):e0271237. doi: 10.1371/journal.pone.0271237 (PMC9278730; doi:10.1371/journal.pone.0271237)
Supplement: S1 File — (PDF) [file pone.0271237.s001.pdf]

Annex 1. Gaaffilee Afaan Oromootiin

**Kutaa A: Odeeffannoowwan dhimma hawaasummaa kee irratti xiyyeeffatan**			
Lakk.	Gaaffiiwwan	Filannoo	Irra Darbi
101	Umuriin kee meeqa?	waggaan ______	

102	Kutaa meeqa baratta?	kutaa ______	
103	Amantaa kee maali?	1. Kiristaana<br>2. Musliima<br>3. Hin qabu<br>4. Kanbiraa_____	
104	Aabboon/Abbaan/ kee barnoota ni qabuu?	1. Dubbisuu fi barreessuu kan hindandeenye<br>2. Dubbisuu fi barreessuu kan hindandeenye<br>3. Barnoota Sad. 1ffaa<br>4. Barnoota Sad.2ffaa<br>5. Dippiloomaa fi isaa ol	
105	Aayyoon/Haati kee barnoota ni qabuu?	1. Dubbisuu fi barreessuu kan hindandeenye<br>2. Dubbisuu fi barreessuu kan hindandeenye<br>3. Barnoota Sad. 1ffaa<br>4. Barnoota Sad.2ffaa<br>5. Dippiloomaa fi isaa ol	
106	Hojiin Aabboo/Abbaa keetii maali?	1. Daldalaa<br>2. Hojjetaa mootummaa<br>3. Hojjetaa mit-mootummaa/dhuunfaa<br>4. Qonnaan bulaa<br>5. Kan biraa yoota'e, barreessi________	
107	Hojiin Aayyoo/Haadha keetii maali?	1. Daldaltuu<br>2. Hojjettuu mootummaa<br>3. Hojjettuu mit-mootummaa/dhuunfaa<br>4. Qonnaan bultuu<br>5. Haadha manaa<br>6. Kan biraa yoota'e, barreessi________	
108	Eenyu waliin jiraataa jirta?	1. Maatii wajjin<br>2. Haadha /Abbaa qofa<br>3. Obboleessa/obboleettii qofa<br>4. Fira/Aantee<br>5. Hiriyaa/hiriyoota<br>6. Kophaa<br>7. Kan biraa______	

109	Bakki dhaloota kee	1. Magaala<br>2. Baadiyyaa	
**Kutaa B: Gaaffii beekumsa kaanserii fiixee Gadameessaa irratti**			
110	Kaanserii fiixee Gadameessaa yeroo jedhamu dhageessee beektaa?	1. Eeyyee<br>2. Lakki	Yoo lakki jette gaaffii 116-ffatti ce'i
111	Kaanseriin fiixee Gadameessaa dubartoota miidhuun beekama	1. Eeyyee<br>2. Lakki<br>3. Hin beeku	
112	Qaamotni Kaanserii fiixee Gadameessaatiin miidhamuu danda'an	1. Gadameessa<br>2. Harma<br>3. Kan biraa<br>4. Hin beeku	
113	Karaan ittiin namni tokko Kaanseriin fiixee Gadameessaatiin qabamuu danda'u/ namaa namatti darbu	1. Karaa quunnamtii saalaa<br>2. Maatii irraa<br>3. Kan biraa<br>4. Hin beeku	
114	Kanneen keessaa kamtu Kaanserii fiixee Gadameessaatiif nama saaxila (*Deebii tokkoo ol kennuu dandeessa*)	1. 'Human papillomavirus" infeekshinii<br>2. Hiriyaa saal-qunnamtii baay'isuu<br>3. Saalquunnamtii ijoollumman eegaluu<br>4. Tamboo aarsuu<br>5. Seenaa dhukkuboota saalqunnamtiin daddarban qabaachuu<br>6. Hin beeku	
115	Mallattoo/wwan Kaanserii fiixee Gadameessaa ibsan<br><br>(*Deebii tokkoo ol kennuu dandeessa*)	1. Marsaa dhiigaa kan laguu/sirrii hintaane jiraachuu<br>2. Dhangala'oo fooli qabeessa kara qaama saalaa dhangala'u<br>3. Mura/dhukkubbii handhuuraa gadii<br>4. Hin beeku	
**Kutaa C: Beekumsa 'Human papillomavirus' infeekshinii fi kittibaata isaa irratti**			
116	'Human papillomavirus' infeekshiniin vaayirasii karaa quunnamtii qaama saalaatiin daddarbu tahuu isaa dhageessee beektaa?	1. Eyyee<br><br>2. Lakki	

117	Eenyutu 'Human papillomavirus' infeekshiniin qabamuu danda'a?	1. Dhiira qofa<br>2. Dhalaa qofa<br>3. Dhiiraa fi dhalaa<br>4. Hin beeku	
118	Dhukkuboota kanniin keessaa kamtu 'Human papillomavirus' infeekshiniin dhufuu danda'a? (***Deebii tokkoo ol kennuu dandeessa***)	1. Kaanserii fiixee Gadameessaa<br>2. Kaanserii qaama saala dhiiraa<br>3. Kaanserii Laagaa<br>4. Kaanserii qaawa bobbaa<br>5. Shiffee qaama saalaa<br>6. Hin beeku	
119	Tooftaalee/ maloota kanniin keessa kamtu'Human papillomavirus' infeekshinii ittisuuf/hirdhisuuf oola? (***Deebii tokkoo ol kennuu dandeessa***)	1. Saalquunnamtii irraa ofqusachuu<br>2. Kittibaata<br>3. Kondomii fayyadamuu<br>4. Ittifamuu hin danda'u<br>5. Hin beeku	
120	'Human papillomavirus' infeekshiniin akka nama qabuuf waantoti haala mijeessan? (***Deebii tokkoo ol kennuu dandeessa***)	1. Hiriyaa Saalqunnamtii baay'isuu<br>2. Tuttuqqii gogaa kan naannoo qaama saalaatti taasifamu<br>3. Dhangala'oo qaamaa(Dhiiga)<br>4. Hin beeku	
**Kutaa D: Beekumsa kittibaata 'Human papillomavirus' infeekshinii irratti**			
121	Kittibaati Human papillomavirus infection dhukkuba infeekshinii 'huumaan paappillooomaa vaayirusiitiin' dhufu ittisuuf akka oolu dhageessee beektaa?	1. Eeyyee<br>2. 2. lakki	Yoo 'hin-dhaga'in' gara gaaffii 126-ffaatti darbi
122	Eenyutu kittibaata'Human papillomavirus' infeekshinii/kaanserii fiixee gadameessaa/ dhorku fudhachuu qaba?	1. Dhiiraa fi dhalaa<br>2. Dhalaa qofa<br>3. Dhiira qofa<br>4. Hin beeku	
123	Waa'ee kittibaata 'Human papillomavirus'	1. Kaanserii fiixee gadameessaa ittisuuf oola	

	infeekshinii/kaanserii fiixee gadameessaa maal beekta?	2. Kaanserii keessoo qaama saalaa/Buqushaa/ ittisuuf oola<br>3. Kaanserii ala qaama saalaa/Buqushaa/ ittisuuf oola<br>4. Hin beeku<br>5. Kan biraa yoo beekte ibsi______________	
124	Kittibaati 'Human papillomavirus' infeekshinii/kaanserii fiixee gadameessaa si'a meeqa kennama?	1. Si'a tokko<br>2. Si'a lama<br>3. Hin beeku	
125	Kittibaati 'Human papillomavirus' infeekshinii/kaanserii fiixee gadameessaa ittisu yoom kennama?	1. Waggaa-9 gaditti<br>2. Waggaa 9–14<br>3. Waggaan hin daangessu<br>4. Hin beeku	
**Kutaa D: Akkaataa hubannoo kaanserii fiixee gadameessaa, 'Human papillomavirus' infeekshinii fi kittibbaata isaa**			
**<u>Carraa qabamuu/Saaxilamuu/Susceptibility</u>**			
126	Dubartiin kamiyyuu 'Human papillomavirus' infeekshinii /kaanserii fiixee gadameessaatiif/ fi miidhaa isaatiif saaxilamtuudha; itti amantaa?	1. Tasallee itti hin amanu<br>2. Itti hin amanu<br>3. Hin amanus hin faallessus<br>4. Haga tokko itti amana<br>5. Baay'een itti amana	
127	Hangam tokko 'Human papillomavirus' infeekshinii /kaanserii fiixee gadameessaatii fi miidhaa isaatiif saaxilamu sodaatta?	1. Tasallee hinsodaadhu<br>2. Hin sodaadhu<br>3. Sodaas/ofitti amantaas hin qabu<br>4. Hanga tokko nin sodaadha<br>5. Baay'een sodaadha	
128	Yoo kittibaata 'Human papillomavirus' infeekshinii /kaanserii balbala gadameessaa hinfudhanne ta'e hangam dhukkubichaaf carraa saaxilamuu qaba jettee yaadda?	1. Tasayyuu carraa saaxilamuu hin qabu<br>2. Carraa saaxilamuu hin qabu<br>3. Qabaachuu fi dhiisuun koo natti hin muldhatu<br>4. Hanga tokko carraa saaxilamuu qabaachuu mala<br>5. Carraa saaxilamuu baay'ee qabaachuu mala	

**Cimina miidhaa dhukkubichaan dhufu/Severity**			
129	‘Human papillomavirus’ infeekshiniin /kaanserii fiixee gadameessaa fi dhukkuboota isaan walqabataniif saaxilamuun jiruu namaa irratti dhiibbaa guddoo geessisa; itti waliigaltaa?	1. Tasayyuu itti walii hin galu<br>2. Itti walii hin galu<br>3. Itti walii hin galus hin mormus<br>4. Ittin waliigala<br>5. Baay’een itti waliigala	
130	‘Human papillomavirus’ infeekshiniin /kaanserii fiixee gadameessaa fi dhukkuboota isaan walqabataniin qabamuun dhukkuba cimaaf nama saaxila; itti waliigaltaa?	1. Tasayyuu itti walii hin galu<br>2. Itti walii hin galu<br>3. Itti walii hin galus hin mormus<br>4. Ittin waliigala<br>5. Baay’een itti waliigala	
131	‘Human papillomavirus’ infeekshiniin /kaanserii fiixee gadameessaa fi dhukkuboota isaan walqabataniin qabamuun baay’ee sukkaneessadha; itti waliigaltaa?	1. Tasayyuu itti walii hin galu<br>2. Itti walii hin galu<br>3. Itti walii hin galus hin mormus<br>4. Ittin waliigala<br>5. Baay’een itti waliigala	
**Bu’aa kittibaata isaa/Benefit**			
132	Kittibaata fudhachuun carraa ‘Human papillomavirus’ infeekshiniif saaxilamuu kee ni hirdhisa	1. Tasayyuu itti walii hin galu<br>2. Itti walii hin galu<br>3. Itti walii hin galus hin mormus<br>4. Ittin waliigala<br>5. Baay’een itti waliigala	
133	Kittibaata fudhachuun miidhaa kaanserii fiixee gadameessaatiin sitti dhufuu malu ni hirdhisa	1. Tasayyuu itti walii hin galu<br>2. Itti walii hin galu<br>3. Itti walii hin galus hin mormus<br>4. Ittin waliigala<br>5. Baay’een itti waliigala	
134	Kittibaaticha fudhachuun shiffee naannoo qaama saalaatti bahu ni hirdhisa	1. Tasayyuu itti walii hin galu<br>2. Itti walii hin galu<br>3. Itti walii hin galus hin mormus<br>4. Ittin waliigala<br>5. Baay’een itti waliigala	
**Haalota akka hin fudhanne gochuu malan/Barriers**			

Kanneeen armaan gaditti eeraman hangam tokko kittibaata kaanserii fiixee gadameessaatiif kennamu akka ati hin fudhanne si dhorkuu malu?			
135	Yaadoo dhiibbaa badaa/side effect/ kittibaatichi geessisuu malu akka ati hin fudhanne si dhorkaa?	1. Baay'ee na dhorka<br>2. Hanga tokko na dhorka<br>3. Na hin dhorkus na hinfudhachiisus<br>4. Na hin dhorku<br>5. Tasayyuu na hin dhorku	
136	Shakkii qulqullinaa fi bu'a qabeessummaa kittibaatichi qabaachuu malu akka ati hin fudhanne si dhorkaa?	1. Baay'ee na dhorka<br>2. Hanga tokko na dhorka<br>3. Na hin dhorkus na hinfudhachiisus<br>4. Na hin dhorku<br>5. Tasayyuu na hin dhorku	
137	Ilaalchi/yaaddoon maatii kee kittibaatichaaf qaban akka ati hin fudhanne si dhorkaa ?	1. Baay'ee na dhorka<br>2. Hanga tokko na dhorka<br>3. Na hin dhorkus na hinfudhachiisus<br>4. Na hin dhorku<br>5. Tasayyuu na hin dhorku	
138	Sodaan ati lilmoo/Marfee waraannachuuf qabdu akka ati hin fudhanne si dhorkaa ?	1. Baay'ee na dhorka<br>2. Hanga tokko na dhorka<br>3. Na hin dhorkus na hinfudhachiisus<br>4. Na hin dhorku<br>5. Tasayyuu na hin dhorku	
139	Hubannoon sirrii hin taane kan ati kittibaatichaaf qabdu akka ati hin fudhanne si dhorkaa ?	1. Baay'ee na dhorka<br>2. Hanga tokko na dhorka<br>3. Na hin dhorkus na hinfudhachiisus<br>4. Na hin dhorku<br>5. Tasayyuu na hin dhorku	
140	Odeeffannoo gahaa dhabuun akka ati hin fudhanne si dhorkaa ?	1. Baay'ee na dhorka<br>2. Hanga tokko na dhorka<br>3. Na hin dhorkus na hinfudhachiisus<br>4. Na hin dhorku<br>5. Tasayyuu na hin dhorku	
**Section E: Ilaalcha 'Human papillomavirus' infeekshinii/ kaanserii fiixee gadameessaa fi kittibaata isaaf qabdu**			
141	Kaanseriin fiixee gadameessaa dhukkuba baay'ee hammaataadha, itti waliigaltaa?	1. Tasayyuu itti walii hin galu<br>2. Itti walii hin galu<br>3. Itti walii hin galus hin mormus<br>4. Hanga tokko ittin waliigala<br>5. Baay'een itti waliigala	
142	Kittibaati dhukkuba kanaaf kennamu carraa dhukkubichaan qabamuu	1. Tasayyuu itti walii hin galu<br>2. Itti walii hin galu<br>3. Itti walii hin galus hin mormus<br>4. Hanga tokko ittin waliigala<br>5. Baay'een itti waliigala	

	hirdhisuuf/dhabamsiisuuf kannama; itti waliigaltaa?		
143	Kittibaati dhukkuboota ittisuuf kennama; itti amantaa??	1. Tasayyuu itti hin amanu<br>2. Itti walii hin amanu<br>3. Itti walii hin amanus hin mormus<br>4. Hanga tokko ittin amana<br>5. Baay'een itti amana	
144	Matiin kee akka ati kittibaata kaanserii fiixee gadameessaa fudhattu fedha qabuu?	1. Tasayyuu hin fedhan<br>2. Hanga tokko hin fedhan<br>3. Fedhii qabaachuu fi dhabuu waanti beekamu hin jiru<br>4. Hanga tokko ni fedhu<br>5. Baay'ee fedhu	
145	Kittibaatichi baay'ee mishaa fi bu'a qabeessadha; itti waliigaltaa?	1. Tasayyuu itti walii hin galu<br>2. Itti walii hin galu<br>3. Itti walii hin galus hin mormus<br>4. Hanga tokko ittin waliigala<br>5. Baay'een itti waliigala	
146	Kittibaaticha fudhachuun barbaachisaa akka ta'ee fi namoota biroon illee akka fudhataniif ni jajjabeessitaa?	1. Tasayyuu hin jajjabeessu<br>2. Hin jajjabeessu<br>3. Hin jajjabeessusus hin mormus<br>4. Hanga tokko nan jajjabeessa<br>5. Baay'een jajjabeessa	
147	Kittibaaticha fudhachuun akka fedhan akka hiriyaa saalquunnamtii baay'isan nama taasisa	1. Baay'een itti waliigala<br>2. Hanga tokko ittin waliigala<br>3. Itti walii hin galus hin mormus<br>4. Itti walii hin galu<br>5. Tasayyuu itti walii hin galu	
**Kutaa F: Madden odeeffannoo fi kaka'umsaa/piroomooshinii/ kittibaata kaanserii fiixee Gadameessaa irratti jirachuu fi dhiisuu isaa**			
148	Waa'ee kittibaata kaanserii fiixee Gadameessaa dhageessee beektaa?	1. Eyyee<br>2. Lakki	Yoo 'Lakki' jette gara 154-ffaatti ce'i
149	Yoo dhageesseetta ta'e eenyurraa dhageesse? (Deebii lamaa ol kennuu dandeessa)	1. Maas-miidiyaa<br>2. Ogeeyyii fayyaa<br>3. Mana barnootaa<br>4. Interneetii<br>5. Hiriyoota kee irraa<br>6. Maatii kee irraa	

		7. Kan biraa yoo jiraate ibsi_______________	
150	Kittibaaticha akka fudhataniif ijoollee kakasuun ni jiraa naannoo kee?	1. Eyyee<br>2. Lakki	
151	Mana barnootaatti waa'ee kittibaata kaanserii fiixee gadameessaa barattee/dhageessee/ beektaa?	1. Eyyee<br>2. Lakki	
152	Ogeeyyiin fayyaa hawaasa naannoo kee keessatti gadi ba'anii(bu'anii) waa'ee kaanserii fiixee gadameessaa barsiisanii beekuu?	1. Eyyee<br>2. Lakki	
153	Warri kittibaaticha kennan osoo hin kennin dursanii odeeffannoo (barnoota) ga'aa waa'ee kittibaatichaa irratti ni kennuu?	1. Eyyee<br>2. Lakki	
154	Waa'ee kittibaata kaanserii fiixee gadameessaa irratti odeeffannoo (barnootni) dabalataa na barbaachisa jettee yaaddaa?	1. Eyyee<br>2. Lakki	**'Lakki'** yoo jette Gaaffii 156-ffaatti ce'i
155	Yoo odeeffannoo (barnooti dabalataa si barbaachisa ta'e, qaama kamiin yoo siif kenname filatta? (**Deebii lamaa ol kennuu dandeessa**)	1. Maas miidiyaa<br>2. Ogeeyyii Fayyaatiin<br>3. Karaa Mana Barnootaa<br>4. Interneetii<br>5. karaa hiriyaa keetiin<br>6. karaa maatii<br>7. kan biraa yoo feete, ibsi________	
**Kutaa G: Gaaffii Kittibaata kaanserii fiixee Gadameessaa fudhachuu kee fi fedhii fudhachuuf qabdu adda baasuuf qophaa'an**			
156	Kittibaata kaanserii fiixee gadameessaa fudhattee beektaa?	1. Eyyee<br>2. Lakki	

			**Lakki’** yoo jette Gaaffii 159-tti ce’i
157	Kittibaata kaanserii fiixee gadameessaa fudhattee beekta yoo ta’e, si’a meeqa fudhatte?	1. 1<br>2. 2	
158	Kittibaata kaanserii fiixee gadameessaa fudhattee beekta yoo ta’e, maaltu akka ati fudhattuuf si gargaare? (***Deebii lamaa ol kennuu dandeessa***)	1. Odeeffannoo gahaa waan argatteef<br>2. Bu’aa isaatti waan amanteef<br>3. Ogeeyyiin fayyaa waan sideeggaraniif<br>4. Maatiin kee akka fudhattu waan si jajjabeessaniif<br>5. Hiriyootni kee akka fudhattu waan si jajjabeessaniif<br>6. Kittibaatichi tolaan waan kennamuuf<br>7. Kan biraa yoo jiraate, ibsi______	
159	Kittibaaticha hin fudhanne yoo tahe, akka ati hin fudhanne maaltu sigodhe? (**Fudhatteeta yoo tahe irra darbi**)	1. Odeeffannoo gahaa waanin hin argatiniif<br>2. Ilaalchi ani kittibaatichaaf qabu sirrii miti<br>3. Guyyaa kittibaati keenamu ani hin turre<br>4. Sodaa miidhaa sann booda dhufuu danda’u irraa maddu<br>5. Soda lilmoo waraannachuu waanin qabuuf<br>6. Maatiin koo akkan ani fudhadhu hin fedhan waanta’eef<br>7. Dhiibbaa Hiriyaa<br>8. Bu’aa isaatti hin amanne<br>9. Hanqina kittibaataatu ture<br>10. Wanta hawaasa keessatti odeeffamu<br>11. Kan biraa yoo jiraate, ibsi____	
