# Supplementary material for: Human papillomavirus vaccination uptake and its associated factors among adolescent school girls in Ambo town, Oromia region, Ethiopia, 2020
Source: PLoS One. 2022 Jul 13;17(7):e0271237. doi: 10.1371/journal.pone.0271237 (PMC9278730; doi:10.1371/journal.pone.0271237)
Supplement: S2 File — (PDF) [file pone.0271237.s002.pdf]

KUTAA-I: Gaaffilee afaaniffaan qaamota odeeffannoo qo'annoo kanaaf dhiyoo jiran irraa sassaabuuf qophaa'e

1. Seensaaf, maqaa fi gahee hojiikee naaf himuu dandeessaa?
2. Tajaajilli beekamaan akka Buufata fayyaa keessaniitti kennamu tokko tajaajila kittibaata daa'immaniiti (kittibaata kaanserii balbala gadameessaas dabalatee). Ati kaampeenii mana barnootaatti kittibaata kaanserii balbala gadameessaaf kennamu irratti hirmaatteetaa?
3. Hubannoo barattootni shamarranii kittibaatichaaf qaban akkamitti ilaalta?
4. Kittibaaticha akka fudhataniif barumsi hubannoo dabaluu(kakaasuu) kennameeraa?
   - Yoo kennameera tahe eenyuun?
   - Eessa fa'itti?
   - Hawaasa baldhaa keessatti gadi bu'amee kennamee beekaa?-yoo hin kennamne tahe maaliifi?
5. Rakkoowwan gurguddoo (ijoo) ta'an kan ati yeroo kittibaatichi kennamu argite maali?
   o Gama ijoolleetiin(barattootaatiin/warra fudhataniitiin)?
   o Gama warra ogeessota faayyaatiin (qindeessitootaatiin)?
6. Prograamichi(sagantichi) akka hin milkoofne waantotni taasisan maali?
7. Waantota (ilaalchota) akka ijoolleen kittibaaticha hin fudhanne godhan kan ati argite maali?
   - Ilaalchota sanniin keessaa kan irra deddeebiin simudate jiraa?
8. Hubannoo fi ilaalchi maatiin isaanii (hawaasni) kittibaatichaaf qaban maal fakkaata?
9. Kittibaaticha waliin waqabatee maaltu fooyya'uu qaba jettee yaadda?
10. Walumaagalatti waanti itti dabaluu barbaaddu yoo jiraate
