# Supplementary material for: Human papillomavirus vaccination uptake and its associated factors among adolescent school girls in Ambo town, Oromia region, Ethiopia, 2020
Source: PLoS One. 2022 Jul 13;17(7):e0271237. doi: 10.1371/journal.pone.0271237 (PMC9278730; doi:10.1371/journal.pone.0271237)
Supplement: S3 File — (PDF) [file pone.0271237.s003.pdf]

**English version Questionnaire on knowledge, attitude, and uptake of human papillomavirus vaccination and its associated factors among adolescent school girls**

| **Section A: information about your socio-demographic characteristics** | | | |
|---|---|---|---|
| S.N | Question and filters | Coding categories | Skip to |
| 101 | How old are you? | In years__________ | |
| 102 | What is your Level of education? | In Grade __________ | |
| 103 | What is your religion? | 1. Orthodox<br>2. Protestant<br>3. Catholic<br>4. Muslim<br>5. No religion<br>6. Other_____ | |
| 104 | What is your fathers' level of education? | 1. Unable to read and write<br>2. Able to read and write<br>3. Primary education<br>4. Secondary education<br>5. College diploma and above | |
| 105 | What is your mothers' level of education? | 1. Unable to read and write<br>2. Able to read and write<br>3. Primary education<br>4. Secondary education<br>5. College diploma and above | |
| 106 | What is the occupation of your father? | 1. Merchant<br>2. Governmental employee<br>3. Private-employee<br>4. Farmer<br>5. If other, specify______ | |
| 107 | What is the occupation of your mother? | 1. Merchant<br>2. Governmental employee<br>3. Private-employee<br>4. Farmer<br>5. House wife<br>6. If other, specify_____ | |
| 108 | With whom you are living? | 1. Together with parents<br>2. Relatives<br>3. With friends<br>4. Alone<br>5. Other _______ | |
| 109 | Your place of birth | 1. Urban<br>2. Rural | |

| Section B: Knowledge of study participant about cervical cancer | | | |
|---|---|---|---|
| 110 | Have you ever heard cervical cancer? | 1. Yes<br>2. No | If No (skip to Q-116) |
| 111 | Cervical cancer is common female cancer | 1. Yes<br>2. No<br>3. I don't know | |
| 112 | Organs affected by cervical cancer | 1. Uterus<br>2. Breast<br>3. I don't know<br>4. Others, specify_____ | |
| 113 | Mode of acquisition of cervical cancer | 1. Sexual transmission<br>2. History of cancer in a family<br>3. I don't know<br>4. Others, specify________ | |
| 114 | Which do you think is/are the risk factor/s for cervical cancer? (more than one answer possible) | 1. Human papillomavirus infection<br>2. Promiscuity<br>3. Early age onset of sexual activity<br>4. Tobacco smoking<br>5. History of sexually transmitted infection<br>6. I don't know | |
| 115 | When do you recognize sign and symptom of cervical cancer? When there is: | 1. Abnormal Vaginal bleeding<br>2. Vaginal discharge<br>3. Dyspareunia<br>4. I don't know | |
| Section C: Knowledge towards HPV infection | | | |
| 116 | Human papillomavirus (HPV) is a virus that causes a sexually transmitted infection, have you ever heard of it? | 1. Yes<br>2. No | |
| 117 | Who can contract HPV infection? | 1. Only men<br>2. Only women<br>3. Men and women<br>4. I don't know | |
| 118 | Which of these diseases are caused by HPV infection? (more than one answer possible) | 1. Cervical cancer<br>2. Penile cancer<br>3. Oropharyngeal cancer<br>4. Anal cancer<br>5. Genital warts<br>6. I don't know | |

| | | | |
|---|---|---|---|
| 119 | Which one do you think that Ways of preventing HPV diseases (more than one answer possible) | 1. Practicing abstinence<br>2. Vaccination<br>3. By using Condoms<br>4. Cannot be prevented<br>5. I don't know | |
| 120 | What are risk factors for HPV infection? ( more than one answer possible) | 1. High frequency of sex partner exchange<br>2. Genital –skin to skin contact<br>3. Body fluids(bloods)<br>4. I don't know | |
| Section D: Knowledge towards HPV vaccination | | | |
| 121 | Human papillomavirus vaccine is a vaccine used to prevent Human papillomavirus infection, have you ever heard of it? | 1. Yes, I have<br>2. No, I haven't | If no, skip to Q-126 |
| 122 | Who should get the HPV vaccination? | 1. Only Men<br>2. Only Women<br>3. Men and women<br>4. I don't know | |
| 123 | The HPV vaccine helps to: | 1. Prevent cervical cancer<br>2. Prevent vaginal cancer<br>3. Prevent vulva cancer<br>4. I don't know<br>5. Other specify______________ | |
| 124 | Recommended doses of HPV-Vaccine | 1. One dose<br>2. Two doses<br>3. I don't know | |
| 125 | The ideal time HPV Vaccine best recommended is: | 1. Less than 9-years<br>2. 9–14 years<br>3. Can be provided at any age<br>4. I don't know | |
| Section E: Perception towards cervical cancer, HPV infection and HPV vaccination | | | |
| <u>Susceptibility</u> | | | |
| 126 | Any women are susceptible to face cervical cancer and victim of its severe health out come through her life, do you believe? | 1. Very untrue of what I believe<br>2. Untrue of what I believe<br>3. Neutral<br>4. True of what I believe<br>5. Very true of what I believe | |

<table>
<tr><td>127</td><td>How much are you worried of contracting HPV infection?</td><td colspan="5">1. Very untrue of me<br>2. Untrue of me<br>3. Neutral<br>4. True of me<br>5. Very true of me</td></tr>
<tr><td>128</td><td>If you don't get vaccinated for HPV, how likely is it that you'll become infected with genital HPV; get cervical cancer; get genital warts in the future?</td><td colspan="5">1. Very unlikely<br>2. Unlikely<br>3. Neither unlikely nor likely<br>4. Likely<br>5. Very likely</td></tr>
<tr><td colspan="7"><u>Severity</u></td></tr>
<tr><td rowspan="2">S.no</td><td rowspan="2">Quastions</td><td colspan="5">Likerts scale Range</td></tr>
<tr><td>Strongly disagree</td><td>Disagree</td><td>Neither disagree nor agree</td><td>Agree</td><td>Strongly agree</td></tr>
<tr><td></td><td>Being infected with HPV; having cervical cancer; having genital warts would have major consequences on life; do you agree?</td><td>☐</td><td>☐</td><td>☐</td><td>☐</td><td>☐</td></tr>
<tr><td>130</td><td>Being infected with HPV; having cervical cancer; having genital warts would be devastating; do you agree?</td><td>☐</td><td>☐</td><td>☐</td><td>☐</td><td>☐</td></tr>
<tr><td>131</td><td>It would be very serious if someone became infected with HPV; had cervical cancer; had genital warts; do you agree?</td><td>☐</td><td>☐</td><td>☐</td><td>☐</td><td>☐</td></tr>
<tr><td colspan="7"><u>Benefit</u></td></tr>
<tr><td>132</td><td>If you get vaccinated for HPV, you can reduce your risk of HPV infection  and cervical cancer</td><td>☐</td><td>☐</td><td>☐</td><td>☐</td><td>☐</td></tr>
<tr><td>133</td><td>If you get vaccinated for HPV, you can reduce the risk of vaginal al cancer</td><td>☐</td><td>☐</td><td>☐</td><td>☐</td><td>☐</td></tr>
</table>

| | | | | | | |
|---|---|---|---|---|---|---|
| 134 | Getting vaccinated for HPV will decrease your chances of getting genital warts | ☐ | ☐ | ☐ | ☐ | ☐ |
| Barriers<br><br>How much the following factors couldn't prevent you from getting vaccinated for HPV? | | | | | | |
| 135 | Concerns about possible side effects | ☐ | ☐ | ☐ | ☐ | ☐ |
| 136 | Concern about safety and effectiveness | ☐ | ☐ | ☐ | ☐ | ☐ |
| 137 | Parental concern | ☐ | ☐ | ☐ | ☐ | ☐ |
| 138 | Needle phobia | ☐ | ☐ | ☐ | ☐ | ☐ |
| 139 | Miss understanding about HPV vaccine | ☐ | ☐ | ☐ | ☐ | ☐ |
| 140 | Lack of information | ☐ | ☐ | ☐ | ☐ | ☐ |
| Section F: Attitude towards HPV vaccination | | | | | | |
| 141 | Cervical cancer is a deadly disease, do you agree? | ☐ | ☐ | ☐ | ☐ | ☐ |
| 142 | The vaccination of the HPV was beginning to minimize [even to terminate] HPV infection and cervical cancer, do you accept? | ☐ | ☐ | ☐ | ☐ | ☐ |
| 143 | Do you think vaccination helps to prevent some diseases? | ☐ | ☐ | ☐ | ☐ | ☐ |
| 144 | May your parents let you have a vaccine? | ☐ | ☐ | ☐ | ☐ | ☐ |
| 145 | HPV vaccine saves life and improve health, do you agree? | ☐ | ☐ | ☐ | ☐ | ☐ |

| | | | | | | |
|---|---|---|---|---|---|---|
| 146 | Do you think that it is important to have vaccination and you recommend others to have HPV vaccination? | ☐ | ☐ | ☐ | ☐ | ☐ |
| 147 | Having the HPV Vaccine could not made you sexually promiscuous | ☐ | ☐ | ☐ | ☐ | ☐ |

| | | | |
|---|---|---|---|
| Section G: Presence of promotion and sources of information about HPV vaccination | | | |
| 148 | Have you ever heard about HPV vaccine before? | 1. Yes, I have<br>2. No I haven't | If No, Skip to Q-154 |
| 149 | If yes, from whom you heard? (More than one answer is possible) | 1. Mass media<br>2. Health Workers<br>3. School<br>4. Internet<br>5. Friends<br>6. Parents/Family<br>7. Other________________ | |
| 150 | Is there any available promotion at your area? | 1. Yes<br>2. No | |
| 151 | Have you been thought about HPV or cervical cancer at school? | 1. Yes<br>2. No | |
| 152 | Do health workers thought you about cervical cancer or HPV through community outreach/other means? | 1. Yes<br>2. No | |
| 153 | Do providers provide full information about HPV Vaccine prior to vaccination? | 1. Yes<br>2. No<br>3. I don't know | |
| 154 | Do you feel you need more information about HPV vaccination? | 1. Yes<br>2. No | If No, Skip to Q-156 |
| 155 | If yes, from whom you prefer? (More than one answer is possible) | 1. Mass media<br>2. Health Workers<br>3. School<br>4. Internet<br>5. Friends | |

| | | | |
|---|---|---|---|
| | | 6. Parents/Family<br>7. Other________ | |
| Section H: HPV vaccine uptake | | | |
| 156 | Have you received HPV vaccination? | 1. Yes, I have<br>2. No; I haven't | If No, skip to Q-159 |
| 157 | If yes, how many doses of the Vaccine? | 1. 1<br>2. 2 | |
| 158 | If you receive, what helps you to receive? (Possibly more than one answer) | 1. Pre information<br>2. Believes on its benefit<br>3. Encouragement from health worker<br>4. Parental influence<br>5. Pear influence<br>6. Because its cost free<br>7. Other, specify_____ | |
| 159 | If you haven't taken HPV vaccine before, why? (skip if you received) | 1. I have no information about the vaccine<br>2. I have negative attitude for the vaccine<br>3. Being absent at vaccination day<br>4. Fear of side effect<br>5. Fear of needle injection<br>6. Parental concern<br>7. Pear influence<br>8. Less believe on its benefit<br>9. Access<br>10. Social pressures/rumors<br>11. Other, specify____ | |

Key: HPV- Human papillomavirus infection
