# Supplementary material for: Human papillomavirus vaccination uptake and its associated factors among adolescent school girls in Ambo town, Oromia region, Ethiopia, 2020
Source: PLoS One. 2022 Jul 13;17(7):e0271237. doi: 10.1371/journal.pone.0271237 (PMC9278730; doi:10.1371/journal.pone.0271237)
Supplement: S4 File — (PDF) [file pone.0271237.s004.pdf]

Question for key informant interview

Interview number _____

Date of interview / /

Interview location ______________________________

Interviewer ______________________________

Time begin: ____________

Time end: ____________

PART-I: ***KII guide for key informants which consisting health professionals, parents, community volunteer workers and teachers (whom expected to have knowledge of the issue on Immunization services/program)***

1. Tell me a little bit about yourself
   a. What is your role at the clinic?
   b. How long have you been working here?
2. One of the prominent services given in this clinic is vaccination program. Have you involved in HPV vaccination in this clinic/ as outreach campaign?
3. Do community outreach for awareness creation available? How do you see it?
4. How do you see awareness of HPV infection and HPV vaccination among adolescents?
5. How would the existence of promotion look likes in relation to the HPV Vaccination services/program?
6. What are the major problem did you observe while HPV vaccination?
7. What are existing barriers for the implementation of the HPV Vaccination services/ program?
8. What barriers did you observe that made adolescents refuse to accept HPV vaccine during vaccination?
   - What factors most repeatedly mentioned?
9. How do you describe perception in relation to HPV vaccination among adolescents and their parental concerns?
10. How do you describe attitude of adolescents and their parents in relation to HPV vaccination?
11. What would you recommend to be improved in relation to HPV vaccination in the feature?

In-depth interview guide for adolescent Girls who are eligible to have HPV vaccination other than those involved in quantitative

"Hello my name is___________ I am from Ambo University and this is my colleague, ____________, who is assisting me.

Thank you so much for giving up some of your time to talk with me today. As you know, the purpose of this interview is to learn about your opinion and experience of vaccination to explore barriers and factors affecting Human papillomavirus vaccination among adolescent Girls in ___________ kebeles of Ambo town.

I am recording our talk, just as a way to take notes, but afterwards, the recording will be transcribed, and the tape will be deleted. No names will be used in the report. So please feel free to speak frankly.

Do you have any questions about the process of this interview?

Would you prefer to speak alone, or can (assistant) remain to observe and make some written notes?"

Interview number _____

Date of interview / /

Interview location ______________________________

Interviewer ______________________________

Time begin: ____________

Time end: ____________

Question for in-depth interview

1. Can you tell me a little bit about yourself?
2. Have you ever heard about the HPV vaccine?
   - If yes, from whom you heard it?
   - If yes, where and what have you heard about it?
3. How do you see adolescents HPV vaccination?
4. Have you received HPV vaccination?
   - If you received, what would influence you to get the HPV vaccine?
   - If you haven't received, what would keep you from getting the vaccine (have you any concern regarding to the vaccine?
   - Had your parents have any concern about the vaccine?
5. What barriers do you think that prevents from taking HPV vaccine?

- How do you see perception of adolescents and their parents in relation to HPV infection and cervical cancer? What is your perception/ how do you perceive HPV infection and cervical cancer?
- How do you see behaviors of adolescents and their parents in relation to HPV vaccination?
- Is there any concern beyond this?

6. Have you ever recommended about HPV vaccination from your parents/friends/others? If you were recommended about it, please specify it________
7. Do you intend to receive HPV vaccination?
   - If yes, why? ____
   - If no, why? ____
8. Would you recommend others?
   □ If Yes, why? ____
   □ If No, why? ____
9. What do you think would be necessarily before vaccination?
10. Is there any gap you feel with HPV vaccination program that you recommend as all?
