# Supplementary material for: Human papillomavirus vaccination uptake and its associated factors among adolescent school girls in Ambo town, Oromia region, Ethiopia, 2020
Source: PLoS One. 2022 Jul 13;17(7):e0271237. doi: 10.1371/journal.pone.0271237 (PMC9278730; doi:10.1371/journal.pone.0271237)
Supplement: S5 File — (PDF) [file pone.0271237.s005.pdf]

INFORMED PARENTAL ASCENT FORM

I invite you and your child to take part in a research study being conducted by Mulugeta W/mariam who is a student at Ambo University College of medicine and health science for his partial fulfillment of MSc, as part of his research study/thesis. The study, as well as your rights as a participant, is described below.

Description: This study will examine adolescents' reactions to Human papillomavirus vaccination among adolescent Girls in ___________ school of Ambo town. Adolescents will be interviewed with structured questionnaires compiled for Human papillomavirus utilization and its associated factors posed by the investigator about their background knowledge, practice, perception and attitude. Your child's interview will be videotaped for use in standard research procedures (e.g. analysis of responses, presentation at professional conferences, etc.) Your child's identity will not be revealed to anyone but the principal investigator(s) and her designated research associates.

Confidentiality: Children's answers will be not associated with their name. Rather, each child will be given an identification number on the interviewer's sheet. The videotape of your child's participation will be destroyed after it has been transcribed.

I agree to the researchers using my child's real name in this research and any publications the results from the research.

____

Signature

I agree to have you audio/videotape my child during this study. I understand this audio/video will only be used for the purposes of research (e.g. analysis of responses, transcriptions of responses, etc.) and will not be available to anyone aside from the researcher:

____

Signature

Risks & Benefits: There are no risks to your child's safety. You may opt to preview the videotape or watch it with your child. The story raises no sensitive or controversial issues and does not contain elements typically frightening to children. Nevertheless, a copy of the videotape has been reviewed by the Ethics Review Board and approved for use in this research. Because the interview engages children in thinking about non-violent conflict resolution, there are potential benefits to your child's ability to handle real-life situations of conflict.

Freedom to Withdraw or Refuse Participation: I understand that my child has the right to stop watching the tape at any time, or to refuse to answer any of the interviewer's questions without prejudice from the investigator.

Grievance Procedure: If I have any concerns or am dissatisfied with any aspect of this study I may report my grievances anonymously if desired to the Human Subjects Institutional Review Board, c/o Dean of post graduate research coordinator office phone Number: ___________________.

Questions? Please feel free to ask the investigator any questions before signing the consent form or at any time during or after the study.

Principal Investigator: Mulugeta W/mariam, Ambo University, college of Medicine and Health Science;
Advisors: Advisors: Gizachew Abdissa (MSc, Assistant professor) and Dr. Eshetu Ejeta (PhD) Ambo University, college of Medicine and Health Science, office phone Number: ____________________.

Informed Consent Statement

I, ______________, give permission for my child, ________________ to participate in the research project entitled, "Knowledge, attitude, human papillomavirus vaccination uptake and its associated factors among Adolescent school Girls in Ambo town." The study has been explained to me and my questions answered to my satisfaction. I understand that my child's right to withdraw from participating or refuse to participate will be respected and that his/her responses and identity will be kept confidential. I give this consent voluntarily.

Parent/Guardian Signature:

___________________________________ ________________________

Signature Date

Investigator Signature:

___________________________________ ________________________

Signature Date
